# Acute Device-Related Thrombus after Watchman Device Implant

**DOI:** 10.1155/2019/8397561

**Published:** 2019-09-03

**Authors:** Muhammad Ajmal, Vijendra Swarup

**Affiliations:** ^1^St. Joseph's Hospital and Medical Center, Phoenix, Arizona, USA; ^2^Cardiology Fellow, Creighton University School of Medicine, Phoenix, Arizona, USA; ^3^Arizona Heart Rhythm Center, Phoenix, Arizona, USA

## Abstract

Atrial fibrillation is characterized by irregularly irregular heart rhythm with an increased morbidity and mortality. It is associated with an increased risk of thromboembolism due to formation of blood clot in the left atrium. Most of these blood clots are formed in the left atrial appendage. The risk of blood clot formation is reduced with the use of anticoagulants. The patients who cannot take anticoagulants due to an increased bleeding risk can undergo percutaneous left atrial appendage (LAA) closure. A Watchman device is used for this purpose. LAA closure with the Watchman device is associated with some adverse effects, and one of them is device-related thrombus. Currently, there are no specific guidelines for the management of device-related thrombus. We present a case of Watchman device-related thrombus which developed 16 hours after the device placement. We will also discuss various options for the management of acute thrombosis.

## 1. Introduction

Atrial fibrillation (AF) is the most common arrhythmia which is strongly associated with thromboembolism. The risk for thromboembolism is determined by the CHA2DS2-VASc score, and this risk is reduced with the use of anticoagulation. But anticoagulants increase the risk of bleeding. Patients with a high CHA2DS2-VASc score and a high risk of bleeding had limited options in the past. But after the successful trial of left atrial appendage (LAA) closure with the Watchman device for nonvalvular AF, now these patients can be managed with occlusion of left atrial appendage [1]. The Watchman device like other prosthetic material used for various cardiac indications is associated with an increased risk of device-related thrombus (DRT) [2]. The incidence of DRT is reported variably in different studies, but acute thrombosis within 24 hours after the device placement has never been reported before.

## 2. Case Report

**Figure 1:**
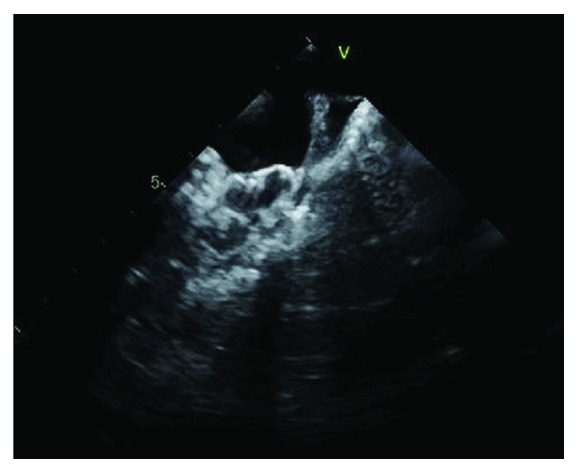
Intraoperative TEE showing a well-positioned Watchman device without thrombus.

A 79-year-old male with a past medical history significant for the long-term persistent atrial fibrillation with a CHA2DS2-VASc score of 4, essential hypertension, hemorrhagic stroke, and dyslipidemia was referred to our electrophysiology clinic for the evaluation of left atrial appendage (LAA) closure. The patient had been taking Warfarin for 10 years, and it was discontinued 3 months ago at the time of cerebral hemorrhage. He was deemed a high risk for the recurrent bleeding. The lab work showed normal complete metabolic panel but had anemia with a hemoglobin level of 9.5 g/dl (N 13.5-17.5 g/dl) on a complete blood count. Because of the high risk for recurrent ischemic stroke and also for bleeding, he was considered a good candidate for the LAA closure with the Watchman device. Transesophageal echocardiogram (TEE) done before the procedure showed mildly dilated left atrium without left atrial thrombus and normal left ventricular and valvular function. The left atrial appendage dimensions were 23 mm osteal diameter and 27 mm depth. He underwent successful placement of the 27 mm Watchman, Boston Scientific device, compressed to a final diameter of 24 mm with a compression factor of 11%. There was no evidence of left atrial thrombus during and after the procedure as shown in Figure 1, and his activated clotting time was measured throughout the procedure which remained therapeutic. There was also no peridevice leak noted on TEE.

**Figure 2:**
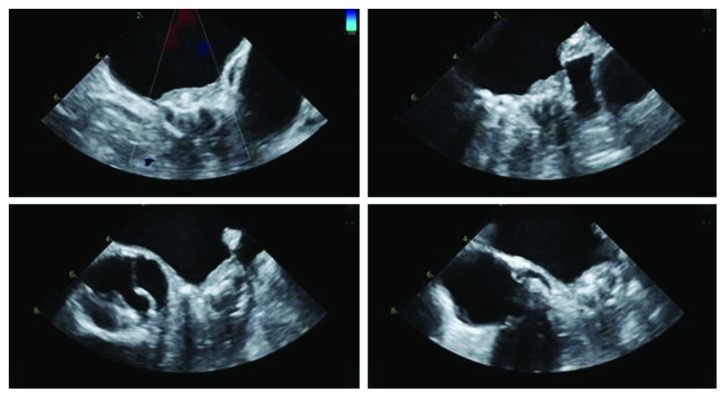
16 hours postoperative TEE showing thrombus on a Watchman device.

After the procedure, Warfarin was started along with aspirin for the prevention of DRT. Next day, he started complaining of chest discomfort and echocardiogram was done to rule out postprocedure complications. Transthoracic echocardiogram showed moderate pericardial effusion which was likely related to device implant. To better visualize the device, transesophageal echocardiogram was done about 16 hours after the procedure and it showed laminar thrombus on the device as shown in Figure 2.

**Figure 3:**
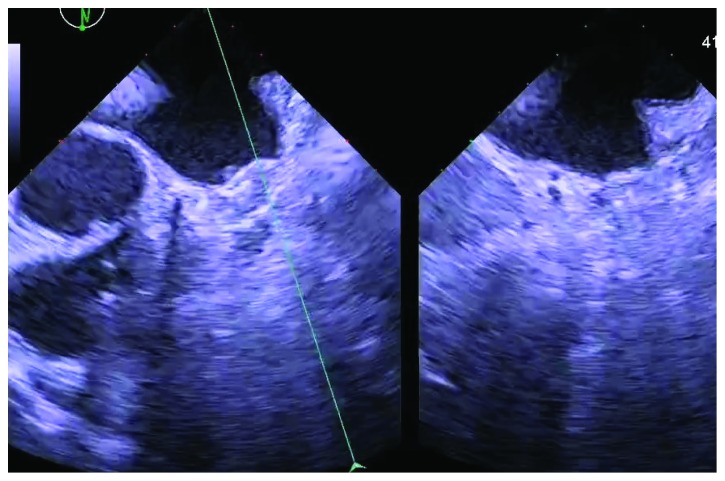
45 days postoperative TEE showing a well-positioned Watchman device without thrombus.

He underwent drainage of about 150 cc of hemorrhagic pericardial effusion. Anticoagulation regimen was not interrupted, and we bridged low molecular weight heparin with Warfarin after the diagnosis of DRT. Heparin bridging was continued until the therapeutic level of international normalized ratio (INR) 2.2 was achieved on day 5. The patient had a close follow-up at clinic in a week after the discharge and did not demonstrate thromboembolic or bleeding complications. We continued the Warfarin with aspirin for 45 days following the procedure. Repeat TEE after 45 days showed a well-positioned device with resolution of thrombus and no peridevice leak as shown in Figure 3. The INR level was therapeutic at 2.3. At this stage, we switched Warfarin to clopidogrel 75 mg daily and continued it with aspirin 81 mg daily until 6 months after procedure. At 6-month follow-up, TEE showed no thrombus and normal functioning device without leak. Clopidogrel was discontinued, and he is kept on lifelong aspirin 81 mg daily.

## 3. Discussion

Atrial fibrillation is associated with an increased risk of stroke and systemic thromboembolism. This risk is reduced with the use of anticoagulation. Traditionally, Warfarin has been used as an anticoagulant in atrial fibrillation to prevent thromboembolism. Although effective, Warfarin has a narrow therapeutic profile, need lifelong monitoring, and has food and drug-drug interactions. It has been reported that approximately 40% eligible patients do not receive anticoagulation, leaving them at a substantial risk for stroke. Echocardiographic and autopsy studies have suggested that left atrial appendage (LAA) is the main source of thromboembolism in patients with AF. The PROTECT AF trial showed noninferiority of left atrial appendage closure with Watchman to Warfarin in the prevention of thromboembolism [3]. US Food and Drug Administration (FDA) approved the Watchman device in March 2015 to reduce the risk of thromboembolism in patients with nonvalvular AF. These patients are at an increased risk for thromboembolism based on the CHADS2 or CHA2DS2-VASC score with an appropriate rationale for seeking nonpharmacological alternative to Warfarin, taking into account the safety and efficacy of the device compared to Warfarin. But these prosthetic devices have some risks too, and one of them is device-related thrombosis (DRT). Ideally, anticoagulation should be continued until complete occluder endothelialization occurs. Anticoagulation regimen after placement of the Watchman device is Warfarin with aspirin for 45 days. After 45 days, Warfarin is switched to clopidogrel after ruling out device-related thrombosis and significant peridevice leak (>5 mm) on TEE. After 6 months, clopidogrel is stopped and patients are kept on lifelong aspirin. Despite using this regimen, thrombus formation on the Watchman device is not uncommon. Predisposing factors for the development of thrombus are multifactorial including patient- and device-related factors [4]. These factors include but are not limited to prior thromboembolism, high CHA2DS2-VASC score, and deeply implanted device or large device [5, 6]. The device-related thrombosis is reported early (at 1.5 months), late (at 3-6 months), and very late (at 12 months). Acute thrombosis within 24 hours has not been reported in literature. The exact etiology for the acute thrombosis is not known but is likely patient related and device related as described above. The management strategy for the device-related thrombosis is challenging as there are no set of guidelines about the choice and duration of anticoagulant. Although classically Warfarin was studied in the trials but as we know, direct oral anticoagulants (DOACs) have been effective in nonvalvular AF and there are small studies which showed promising results after Watchman device placement. Although it needs further research to explore the duration and choice of anticoagulants for DRT, we have few options to prevent the acute thrombosis. The literature search has showed that patients with a high CHA2DS2-VASC score have a high risk for DRT so the anticoagulation regimen should be individualized accordingly. Patients who are at a high risk for thrombosis and lower risk for bleeding should be bridged with parenteral anticoagulants when using Warfarin, as Warfarin can take a few days to be therapeutic. The bridging can be done by using intravenous heparin if the patient is admitted in the hospital or with low molecular weight heparin if the patient is discharged. Second option is the use of DOACs which will be effective in preventing acute DRT because of their rapid onset of action [7]. In our patient, we bridged it with low molecular weight heparin till the INR was therapeutic.

## 4. Conclusion

Percutaneous closure of left atrial appendage for AF has a risk of device-related thrombosis. This complication can occur at any time after the procedure, and the shortest duration as reported in our case can be a few hours. Although it was an incidental finding in our case, it demonstrates the importance of follow-up imaging in days rather than weeks (45 days) especially for the patients who are at a high risk for thrombosis. The real challenge is the choice and duration of anticoagulation for acute DRT. We propose that acute thrombosis should be treated either with Warfarin bridged with heparin until the INR is therapeutic or with DOACs which have rapid onset of action. The duration of anticoagulation should be based on the resolution of thrombus on follow-up imaging. Future studies will be helpful in the management of DRT.
